# Pathophysiology, evaluation, and management of malnutrition in hematologic malignancies: a comprehensive review

**DOI:** 10.3389/fnut.2026.1777896

**Published:** 2026-03-09

**Authors:** Mengting Yang, Xushu Zhong, Yutong He, Yang Dai, Qiaolin Zhou, Wenyi Liang, Zhuohang Zou, Yushuang Xiao, Luocheng Zhang, He Li, Ailin Zhao, Ting Niu

**Affiliations:** 1 Department of Hematology, Institute of Hematology, and Center for High Altitude Medicine, West China Hospital, Sichuan University, Chengdu, China; 2 West China Medical School, West China Hospital, Sichuan University, Chengdu, China; 3 State Key Laboratory of Biotherapy, Collaborative Innovation Center of Biotherapy, West China Hospital, Sichuan University, Chengdu, China; 4 National Facility for Translational Medicine (Sichuan), West China Hospital, Sichuan University, Chengdu, China; 5 Chengdu University of Traditional Chinese Medicine, Chengdu, China

**Keywords:** hematologic malignancies, hematopoietic stem cell transplantation, immunometabolism, malnutrition, nutritional assessment

## Abstract

Hematologic malignancies are frequently complicated by malnutrition, a condition that remains underrecognized yet strongly associated with impaired treatment tolerance, immune recovery, and survival. Unlike solid tumors, hematologic malignancies are characterized by diffuse marrow and immune system involvement, rendering host metabolism highly vulnerable to tumor-driven inflammation and therapy-induced immune stress. Accumulating evidence indicates that nutritional deterioration in hematologic malignancies reflects a state of integrated immunometabolic vulnerability—driven by hyperinflammation, anabolic resistance, gastrointestinal injury, and psychosocial stress—rather than inadequate caloric intake alone. This review synthesizes current evidence on the biological basis, clinical assessment, and management of malnutrition in hematologic malignancies, with particular emphasis on hematopoietic stem cell transplantation and cellular therapies. We propose that nutritional care in hematologic malignancies should evolve from supportive supplementation toward mechanism-informed, precision nutritional strategies aimed at modulating host metabolism and immune function to improve clinical outcomes.

## 1 Introduction

Hematologic malignancies (HM) encompass a heterogeneous group of cancers—including leukemias, lymphomas, and myelomas—that collectively account for a substantial and growing proportion of global cancer incidence. In 2022, there were over 1.3 million new cases of hematologic neoplasms worldwide, constituting 6.5% of the 36 cancers investigated, and over 750,000 deaths, representing 7.2% of cancer deaths (1). While advances in molecular diagnostics, targeted therapies, and immunomodulatory agents have markedly improved overall survival (OS), patient outcomes remain variable. Treatment-related toxicity and disease-associated cachexia continue to contribute substantially to morbidity and mortality.

A critical, yet often underappreciated determinant of these adverse outcomes is the high prevalence of malnutrition and cachexia among HM patients. In a cohort of hospitalized adults with HM, 25.8% met the Global Leadership Initiative on Malnutrition (GLIM) criteria for malnutrition at admission (2). Pediatric studies echo this prevalence, with 28.4% of children presenting with a high risk of malnutrition at diagnosis and 13.7% already underweight (BMI < 5th percentile) (3). The etiology of malnutrition in this population is multifactorial. Therapeutic toxicities frequently result in gastrointestinal dysfunction, including delayed gastric emptying, dysmotility, intolerance to enteral feeding, and malabsorption (2). Concurrently, the malignancy itself often induces a catabolic state, exacerbating protein-calorie malnutrition (4). The clinical impact of this nutritional vulnerability is profound. Malnourished patients exhibit reduced tolerance to intensive chemotherapy regimens, heightened susceptibility to infections, prolonged hospitalization, and increased therapeutic costs (2, 5–7).

Consequently, effective nutritional intervention is paramount for patients with HM. Systematic nutritional support enhances tolerance to intensive chemotherapy and radiotherapy, attenuates toxicities, and minimizes treatment interruptions or dose modifications (8). Furthermore, adequate energy and protein provision preserves body weight and skeletal muscle mass, inhibiting cachexia progression and establishing a foundation for improved quality of life and OS (9, 10). Integrating nutritional support into comprehensive treatment regimens is therefore essential for optimizing clinical outcomes.

In this review, we synthesize current evidence on the prevalence and biological basis of malnutrition in HM, critically evaluate contemporary tools for nutritional assessment and prognostic stratification, and summarize available nutritional intervention strategies. By reframing malnutrition as a dynamic immunometabolic state rather than a simple deficit in intake, we aim to provide a conceptual framework for precision nutritional interventions tailored to the unique vulnerabilities of patients with HM.

To provide an integrative overview of current evidence, this narrative review was informed by a structured literature search conducted in PubMed, Web of Science, and Embase databases, including studies published in English up to December 2025. Search terms included “hematologic malignancies,” “malnutrition,” “nutritional assessment,” “enteral nutrition,” “parenteral nutrition,” “hematopoietic stem cell transplantation”, and related keywords. Original studies, high-quality systematic reviews, and relevant consensus guidelines were included to inform this narrative review.

## 2 Nutritional status and clinical significance in hematologic malignancies

### 2.1 Prevalence and prognostic implications of malnutrition in hematologic malignancies

Nutritional abnormalities are prevalent among patients with HM and closely linked to adverse clinical outcomes. Calleja et al. demonstrated that the prevalence of malnutrition among hospitalized hematology-oncology patients at admission was 47.7%, which was significantly associated with higher readmission rates compared to well-nourished patients (11). A systematic review of 13 studies further confirmed the association between malnutrition and poor prognoses, including reduced OS, increased transplant-related mortality, and an elevated risk of relapse (12). Specifically, patients with severe malnutrition—particularly those with low body mass index (BMI) or concurrent sarcopenia—exhibit poorer tolerance to chemotherapy and pronounced delays in neutrophil recovery (13). This burden is particularly severe in the elderly population with HM; studies indicate that 43% of elderly patients are at risk of malnutrition, with 15% already diagnosed, which serves as an independent predictor of poor outcomes (14).

Beyond undernutrition, body composition and inflammatory markers play a critical prognostic role. Overweight status is also prevalent among patients with HM prior to chemotherapy; however, these patients often present with nutritional inflammatory risk, characterized by hypoalbuminemia, elevated C-reactive protein (CRP) levels, and a high CRP/albumin ratio (15). This inflammatory-nutritional imbalance may impair chemotherapy tolerance and negatively influence prognosis. Research has confirmed that the CRP/albumin ratio (CAR) serves as a highly valuable prognostic biomarker, particularly in elderly patients undergoing allogeneic hematopoietic stem cell transplantation (allo-HSCT) (16).

HSCT presents significant nutritional challenges both during the conditioning phase and throughout recovery. Malnutrition is a frequent consequence of HSCT, contributing to clinical deterioration. Retrospective analyses show that significant weight loss occurs post-transplantation, accompanied by marked reductions in grip strength, knee extensor strength, and extremity circumference. Laboratory parameters often reflect this deterioration, with decreased serum albumin and increased CRP levels indicating impaired muscle morphology and an enhanced inflammatory response (17). Severe malnutrition, diagnosed via Subjective Global Assessment (SGA), increases the risk of acute graft-versus-host disease (GVHD) and negatively impacts survival (18). Indeed, mortality rates for patients with high nutritional risk undergoing allo-HSCT are at least twice those of lower-risk patients (19). Specific metabolic shifts such as refeeding hypophosphatemia (RH) also occur in up to 78% of HSCT patients, warranting daily serum phosphorus monitoring for early detection (20).

In addition to macronutrient imbalances, micronutrient deficiencies are equally significant and should not be overlooked. Vitamin D insufficiency is common in patients undergoing HSCT and is associated with decreased survival and a higher incidence of chronic GVHD (21–23). Deficiency in 25-hydroxyvitamin D [25 (OH)D] is particularly prevalent in lymphoid malignancies and multiple myeloma (MM), where low levels serve as predictive indicators of disease progression (24–27). Notably, supplementation with calcitriol following HSCT may promote absolute lymphocyte count recovery and prolong relapse-free survival (28). Similarly, vitamin C status declines significantly following myeloablative chemotherapy. The nadir of vitamin C levels is closely coincident with the onset of febrile neutropenia and synchronizes with the peaks of the inflammatory marker CRP and the lipid oxidation marker thiobarbituric acid reactive substances (TBARS), demonstrating a significant inverse correlation (29). Even when corrected prior to conditioning, vitamin C deficiency remains associated with an elevated incidence of acute GVHD and increased long-term mortality (30). Deficiencies in other micronutrients, such as selenium, have also been identified as independent predictors of poor outcomes in pediatric patients with HM (31).

Finally, metabolic disorders, particularly hyperglycemia, represent a major clinical concern. Patients with HM are predisposed to hyperglycemia due to frequent glucocorticoid exposure, immunosuppressants (e.g., tacrolimus), total parenteral nutrition, and physiological stress (32). This condition is closely associated with multiple adverse outcomes, including increased infection risk, organ dysfunction, shortened remission duration, elevated GVHD incidence, and heightened mortality (33, 34).

Overall, patients with HM face complex, intertwined nutritional and metabolic challenges. These factors collectively constitute key determinants of treatment tolerance and complication risk, ultimately exerting a profound impact on patient survival. Importantly, accumulating evidence suggests that malnutrition in HM should not be viewed merely as a secondary consequence of reduced intake, but rather as an integrated clinical phenotype reflecting tumor burden, systemic inflammation, and treatment-induced metabolic stress. In this context, nutritional deterioration both mirrors disease severity and actively amplifies vulnerability to therapy-related toxicity, immune dysfunction, and impaired tissue repair (35). This bidirectional relationship may partially explain why conventional caloric supplementation alone often fails to reverse adverse outcomes, underscoring the need to contextualize nutritional status within the broader biological landscape of HM.

### 2.2 Pathophysiological basis of malnutrition in hematologic malignancies

Malnutrition is a prevalent yet frequently underestimated condition among patients with HM. These patients face unique nutritional challenges stemming from tumor-related factors (such as a hypermetabolic state caused by high tumor burden), treatment-related factors (such as severe mucosal barrier damage from intensive chemotherapy regimens, and GVHD associated with HSCT) as well as psychological burden imposed by the disease. Unlike solid tumors, HM are characterized by diffuse systemic involvement of the bone marrow, peripheral blood, and reticuloendothelial organs, rendering host metabolism particularly susceptible to tumor-driven perturbations. The hematopoietic system itself is highly metabolically active and exquisitely sensitive to nutrient availability, inflammatory signaling, and oxidative stress (36). Consequently, nutritional derangements in HM frequently arise early, evolve rapidly during treatment intensification, and exert disproportionate effects on immune reconstitution and hematopoietic recovery, distinguishing this population from patients with localized solid tumors.

#### 2.2.1 Tumor-driven metabolic and inflammatory perturbations

The malignant process itself represents the primary initiating factor driving malnutrition in patients with HMs. Tumor-induced metabolic hijacking constitutes a central mechanism underlying nutritional deterioration in this population. Metabolic reprogramming is a hallmark of HMs that supports tumorigenesis and survival by altering nutrient usage (36). To sustain uncontrolled proliferation, hematopoietic tumor cells undergo profound metabolic reprogramming, reshaping glucose, amino acid, and lipid metabolism to secure energy and biosynthetic substrates (37, 38). Specifically, reliance on aerobic glycolysis (the Warburg effect) leads tumor cells to consume large amounts of glucose with inefficient ATP yield, which has been proposed to contribute to systemic metabolic stress and increased energy demand (39). In HMs, diffuse involvement of the bone marrow and reticuloendothelial organs may further amplify tumor-driven metabolic burden at the systemic level. Concurrently, HMs provoke a pronounced and sustained systemic inflammatory response. Tumor cells and their microenvironment secrete high levels of pro-inflammatory cytokines, including TNF-*α*, IL-1, and IL-6, resulting in sustained systemic inflammation affecting multiple organs and intersects with metabolic pathways linked to cachexia (40). This inflammatory milieu triggers severe tissue depletion, including accelerated myofibrillar protein degradation and increased lipolysis, ultimately leading to anabolic resistance in which dietary intake is inefficiently converted into body tissue (41, 42). At the molecular level, anabolic resistance is mediated by impaired insulin and IGF-1 signaling, suppression of mTOR activity, and mitochondrial dysfunction within skeletal muscle and other metabolically active tissues. As a result, even adequate nutritional intake fails to restore lean body mass in the face of ongoing systemic metabolic and inflammatory stress, contributing to the refractory nature of cancer-associated malnutrition in HM (39). This integrated metabolic and inflammatory phenomenon provides a mechanistic rationale for the limited efficacy of conventional nutritional supplementation strategies unless inflammation and metabolic stress are also concomitantly addressed. Together, these metabolic and inflammatory mechanisms provide a fundamental explanation for the difficulty of correcting malnutrition in patients with HMs.

Beyond systemic metabolic and inflammatory effects, direct gastrointestinal infiltration further compromises intestinal integrity. Infiltration occurs in 25–50% of leukemia patients, particularly during relapse, and approximately 10% of non-primary intestinal lymphoma cases (43, 44). Plasma cell disorders like MM may also involve multiple gastrointestinal segments (45). Such infiltration can result in obstruction, ulceration, hemorrhage, and protein-losing enteropathy. Furthermore, tumor infiltration of the liver and biliary tract—common in lymphomas and myeloid leukemias—impairs lipid and vitamin absorption (46). Additionally, immune dysfunction increases the risk of common and opportunistic gastrointestinal infections, such as necrotizing enterocolitis, esophageal candidiasis, or cytomegalovirus infection (47).

#### 2.2.2 Treatment-related toxicities and immunometabolic stress

Second, treatment-related adverse events represent a key factor exacerbating malnutrition in patients with HM. Conventional therapies, including high-dose chemotherapy, radiotherapy, and HSCT conditioning, damage rapidly proliferating epithelial cells. This epithelial injury results in mucositis, malabsorption, diarrhea, and pain that severely restrict oral intake and nutrient absorption (48–50).

Pharmacological toxicities further complicate nutritional status during intensive anti-cancer treatment. Platinum-based and alkylating agents frequently induce dysgeusia, which suppresses appetite and reduces food intake (51). Additionally, opioids used for pain management frequently induce constipation and delayed gastric emptying, limiting effective digestion and nutrient utilization. Early after allo-HSCT, immunosuppressive agents such as cyclosporine and tacrolimus, cause renal magnesium wasting and hypomagnesemia independent of intake, as demonstrated by Aisa et al. (52). Given the central role of magnesium in cellular metabolism and lymphocyte function, treatment-induced hypomagnesemia may contribute to impaired immune reconstitution and increased post-transplant complications. After allo-HSCT, extensive antibiotic exposure induces profound intestinal microbiota dysbiosis, impairing microbial-mediated nutrient metabolism and epithelial integrity (53, 54). This dysbiosis exacerbates malnutrition and contributes to immune dysregulation and GVHD severity.

In the post-HSCT setting, patients face distinct challenges; severe mucosal inflammation and gastrointestinal GVHD (55, 56) can lead to chronic diarrhea and exocrine pancreatic insufficiency, driving significant weight loss (57). Emerging immunotherapies introduce novel nutritional risks. Chimeric antigen receptor T-cell (CAR-T) therapy, as a salvage treatment for refractory HM, is frequently complicated by cytokine release syndrome (CRS). The activation of CAR-T cells releases pro-inflammatory cytokines, resulting in gastrointestinal adverse effects (58), systemic hypermetabolism, and prominent muscle catabolism, the latter often worsened by glucocorticoids used to treat CRS (59). Notably, the severity of CRS has been identified as an independent risk factor for hypoalbuminemia (60). Similarly, tyrosine kinase inhibitors (TKIs) are associated with gastrointestinal adverse effects in a subset of patients, further complicating nutritional management (61).

Beyond direct gastrointestinal toxicity, modern therapies for HM profoundly reshape host immunometabolism. Intensive cytotoxic regimens, immune checkpoint modulation, and cellular therapies induce acute inflammatory states that simultaneously increase resting energy expenditure and suppress anabolic pathways (62). This immune–metabolic coupling is particularly pronounced in the settings of CRS and GVHD, where sustained cytokine exposure accelerates muscle proteolysis and hypoalbuminemia, thereby aggravating nutritional decline and delaying functional recovery.

#### 2.2.3 Psychosocial stress and neuro–immune–metabolic dysregulation

Finally, psychosocial factors play a non-negligible role in the pathogenesis of malnutrition among patients with HM. Psychological states, including anxiety, depression, and chronic stress, are consistently associated with reduced appetite, altered eating behaviors, and inadequate dietary intake in patients with cancer, including those with HMs (63). Physiologically, anxiety and depression activate the hypothalamic–pituitary–adrenal (HPA) axis and sympathetic nervous system, leading to sustained elevations in circulating stress hormones and pro-inflammatory cytokines, including IL-6 and TNF-*α* (64). These cytokines act on hypothalamic nuclei to stimulate anorexigenic pathways while suppressing orexigenic signaling, thereby promoting early satiety and reduced food intake (65). Chronic stress further enhances sympathetic tone, which can impair gastrointestinal motility, delay gastric emptying, and promote symptoms such as nausea and dyspepsia, thereby indirectly limiting nutrient intake. In the hematology setting, prolonged protective isolation to prevent infection is common and may exacerbate social isolation and loneliness, factors that have been linked to appetite suppression and reduced food intake (66). The overall disease burden—comprising frequent hospitalizations, invasive treatments, and financial stress—creates a vicious cycle: low mood reduces nutrient intake, while malnutrition aggravates fatigue and depression. Accordingly, international supportive care guidelines advocate routine psychological screening and timely intervention as integral components of comprehensive nutritional care in patients with cancer, including those with hematologic malignancies (67). Notably, psychosocial stress does not merely reduce dietary intake, but also perpetuates a state of low-grade systemic inflammation through sustained activation of the hypothalamic–pituitary–adrenal axis and sympathetic nervous system (68). This neuro–immune–metabolic axis may synergize with tumor- and treatment-related inflammatory pathways, amplifying anorexia, anabolic resistance, and muscle wasting in patients with HMs.

**Figure 1:**
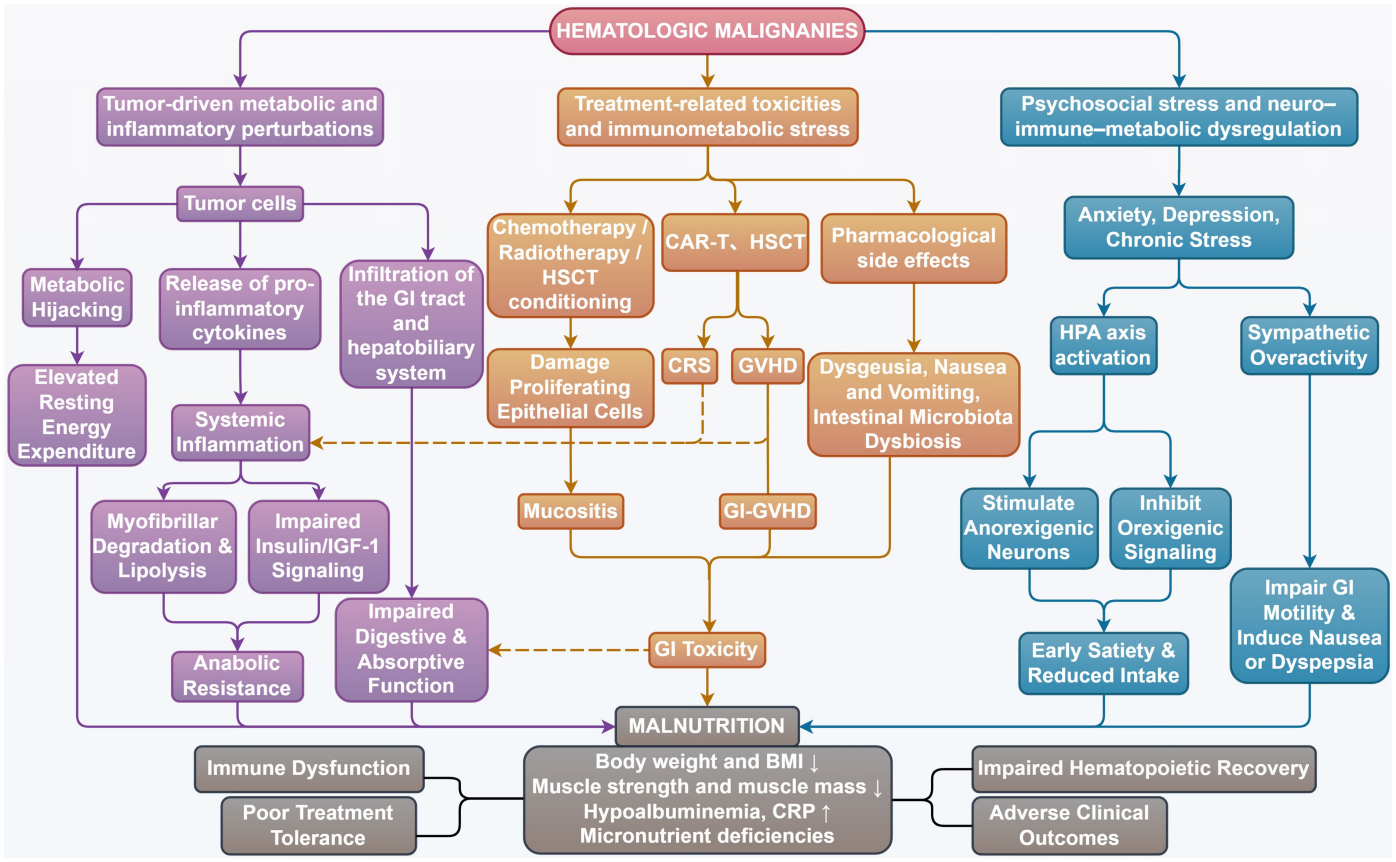
Pathophysiological network underlying malnutrition in hematologic malignancies. Malnutrition in hematologic malignancies arises from the integrated effects of tumor-driven metabolic and inflammatory perturbations, treatment-related toxicity and immunometabolic stress, and psychosocial neuro–immune-metabolic dysregulation, ultimately leading to impaired nutritional status, immune dysfunction, reduced treatment tolerance, and adverse clinical outcomes.

Collectively, malnutrition in HMs emerges from the convergence of tumor-driven hypermetabolism, treatment-related toxicity, and psychosocial stress, forming an integrated pathophysiological network rather than isolated contributors (Figure 1). Accordingly, effective nutritional interventions require a mechanism-informed, multidisciplinary approach that integrates metabolic, inflammatory, and psychosocial dimensions to meaningfully improve clinical outcomes.

## 3 Assessment of nutritional status and prognostic stratification in hematological malignancies

To develop optimal nutrition intervention strategies for patients with HM, accurate determination of their nutritional status is prerequisite for delivering precise nutrition support. In clinical practice, nutritional diagnosis follows a stepwise framework consisting of nutritional risk screening, formal nutritional assessment and malnutrition diagnosis, and subsequent comprehensive metabolic and functional evaluation.

### 3.1 Nutritional screening

The primary purpose of nutritional screening is to rapidly identify HM patients who are at increased nutritional risk and therefore require further comprehensive nutritional assessment, rather than to establish a definitive diagnosis of malnutrition.

Various standardized tools are employed depending on the clinical context. The Nutritional Risk Screening 2002 (NRS-2002) (69) is commonly used for general risk screening. For identifying specific malnutrition risk, preferred tools include the Malnutrition Universal Screening Tool (MUST) (70, 71), Malnutrition Screening Tool (MST) (72), Nutritional Risk Index (NRI) (73), and the Mini Nutritional Assessment-Short Form (MNA-SF). Comparative studies in hospitalized HM patients have shown that MUST may be more suitable than MST, as validated against the Subjective Global Assessment (SGA) (74).

Screening strategies must be adapted for specific patient subgroups. The modified Nutrition Risk in the Critically Ill (mNUTRIC) score and NRS-2002 are effective for HM patients in the ICU, with one study identifying high nutritional risk in 48.4 and 54.4% of patients, respectively. High risk identified by NRS-2002 is significantly associated with increased ICU mortality (75). Additionally, the Geriatric Nutritional Risk Index (G-NRI), calculated using serum albumin and ideal body weight, has prognostic value (76); severe malnutrition (NRI < 83.5) is a predictor of mortality in patients with HM admitted to the ICU due to acute respiratory failure (73). The Graz Malnutrition Screening (GMS) tool, which evaluates weight loss, BMI, food intake, and disease severity, has been applied to predict nutritional risk in patients prior to HSCT. This tool identified approximately 10% of HM patients as being at high nutritional risk (77). For pediatric HM patients, the STRONGkids and PYMS, two validated pediatric nutritional risk screening tools, provide reliable screening criteria for clinical nutritional management (3). The NUTRISCORE is a newly developed instrument for oncological patients (including HM) designed to improve accuracy by integrating traditional parameters with disease-specific factors such as lesion extent and treatment regimens. It shows potential as a simple, effective alternative to tools like the MST (78). An overview of commonly used nutritional screening tools in patients with HM, including their key parameters, clinical settings, and reported performance, is summarized in Table 1.

**Table 1:** Nutritional screening tools used in patients with hematologic malignancies.

Tool	Purpose	Key parameters	Clinical setting	Main findings in HM	Limitations
NRS-2002	Nutritional risk screening	BMI, weight loss, intake, disease severity	Hospitalized adult patients	High NRS-2002 is significantly associated with increased ICU mortality (75).	Lacks objective assessment of body composition and inflammatory status.
MUST	Malnutrition risk screening	BMI, unintentional weight loss, acute disease effect (no intake >5 days)	Community and hospitalized adult patients	MUST has been shown to identify a substantial proportion of hospitalized HM at nutritional risk (71).	Less sensitive to disease severity and inflammatory burden.
MST	Malnutrition risk screening	Unintentional weight loss, reduced appetite	Hospitalized adult patients	MST is feasible for initial nutritional screening in HM inpatients (85).	Diagnostic accuracy is reduced during prolonged hospitalization, with markedly decreased sensitivity compared with MUST.
NRI	Malnutrition risk screening	Serum albumin, current-to-usual body weight ratio	Hospitalized adult patients	NRI-based nutritional risk stratification strongly correlated with adverse ICU outcomes of HM patients, including mortality and prolonged ICU stay (73).	Influenced by inflammatory status.
MNA-SF	Malnutrition risk screening	Recent food intake, weight loss, mobility, psychological stress or acute disease, neuropsychological problems, BMI or calf circumference	Older adult patients (community and hospitalized)	Most of the older patients with an aggressive HM are at risk for malnutrition detected through the MNA-SF (147).	Primarily validated in geriatric populations
GMS	Malnutrition risk screening	Weight loss, BMI, reduced food intake, disease severity	Hospitalized adult patients	GMS-based malnutrition risk stratification before HSCT was associated with a more than twofold increase in mortality risk, particularly in allogeneic transplant recipients (77).	Does not incorporate disease-specific inflammatory burden
mNUTRIC	Nutritional risk screening	Age, APACHE II score, SOFA score, number of comorbidities, days from hospital to ICU admission	Critically ill adult patients (ICU)	The mNUTRIC score did not independently predict ICU mortality in patients with HM (75).	Not designed for non-ICU patients
G-NRI	Malnutrition risk screening	Serum albumin, current-to-ideal body weight ratio	Older adult patients (hospitalized and outpatient)	Lower G-NRI values were associated with significantly worse survival outcomes, even after adjustment for transplant-related risk factors (76).	Influenced by inflammatory status
STRONGkids	Nutritional risk screening	Underlying illness with malnutrition risk, subjective clinical assessment, manifestation assessment, weight loss or poor weight gain	Hospitalized pediatric patients	STRONGkids is useful for early identification and longitudinal monitoring of nutritional risk in pediatric HM patients (3).	Lacks objective assessment of body composition and inflammatory status
PYMS	Nutritional risk screening	BMI centile, recent weight loss, recent change in nutritional intake, effect of current illness on nutrition	Hospitalized pediatric patients	PYMS identified a higher proportion of pediatric HM patients at nutritional risk than BMI alone (3).	Lacks objective assessment of body composition and inflammatory status
NUTRISCORE	Nutritional risk screening	Tumor site, recent weight loss, reduced food intake, oncologic treatment	Adult patients with cancer	/	Lacks objective assessment of body composition and inflammatory status

Although traditionally categorized as assessment parameters, body weight is frequently used at the screening stage in routine clinical practice, particularly in resource-limited or high-acuity settings. Body weight and associated factors remain the simplest and most common assessment tools, yet their interpretation requires nuance. A meta-analysis demonstrated that patients with HM who have a low BMI before HSCT and during HSCT exhibit significantly shorter OS and EFS (79). While weight loss and low body weight are widely recognized indicators of malnutrition (80), their prognostic interpretation in HM is highly dependent on the classification method used. In the context of HSCT, age- and gender-adjusted BMI has emerged as a superior prognostic tool compared to WHO BMI or ideal body weight classifications. Adjusted BMI effectively predicts OS, progression-free survival (PFS), and relapse rates, underscoring the importance of accounting for demographic variables (81).

Obesity generally correlates with adverse outcomes, including increased non-relapse mortality (NRM), infection risk, and complications in allo-HSCT (82). However, a retrospective study revealed that while high adjusted BMI increased NRM and acute GVHD risk, it markedly reduced relapse risk, resulting in no significant difference in OS compared to normal BMI. Conversely, low adjusted BMI consistently predicts inferior outcomes (81). Notably, in critically ill HM patients, obesity and malnutrition frequently coexist, rendering BMI potentially misleading. Malnutrition defined by combined criteria (BMI, ICD-10, ASPEN) correlates with mortality, whereas isolated obesity (BMI ≥ 30 kg/m^2^) does not (83). These findings highlight that body weight alone reflects nutritional phenotype rather than biological risk, underscoring the need for integrative assessment approaches.

### 3.2 Nutritional assessment

While screening identifies risk, the core objective of nutritional assessment is to establish the presence and severity of malnutrition and to inform individualized nutrition support strategies. Given the high false-positive rates of screening in oncology populations, routine comprehensive assessment is essential.

Although specific criteria tailored solely to HM are lacking, several tools are widely applied. The Subjective Global Assessment (SGA) and its patient-generated counterpart (PG-SGA) are often considered gold standards (84, 85). They comprehensively evaluate weight changes, intake, symptoms, and physical findings. PG-SGA has successfully demonstrated the negative nutritional impact of high-dose conditioning in autologous PBSCT (86). The Global Leadership Initiative on Malnutrition (GLIM) framework diagnoses malnutrition based on phenotypic (weight loss, low BMI, sarcopenia) and etiologic (intake, inflammation) criteria. In one study, while NRS-2002 identified high risk in 81.7% of HM patients, GLIM criteria diagnosed malnutrition in 25.8%, highlighting the distinction between risk and diagnosis (2). This discrepancy emphasizes that nutritional screening tools should not be used interchangeably with diagnostic frameworks when guiding clinical decision-making. The Mini Nutritional Assessment (MNA) score has been validated as an independent correlate of mortality risk in older adults with HM (87).

Several scoring systems integrate nutritional and inflammatory markers to predict outcomes. The Controlling Nutritional Status (CONUT) score, calculated from albumin, cholesterol, and lymphocyte count, is negatively correlated with OS in HM patients (88), including those with MM (89). A modified version (mCONUT) incorporating CRP is effective for predicting CRS in relapsed/refractory AML patients undergoing CLL1 CAR-T therapy (90). By integrating CRP and albumin, the Glasgow Prognostic Score (GPS) assesses the inflammatory-nutritional status and significantly predicts length of hospital stay in patients with HM (91). The Nutritional and Immune-Inflammatory Scoring System (NII) combines indicators like NRS-2002 and GNRI. An NII score of ≥6 serves as an independent high-risk prognostic factor in diffuse large B-cell lymphoma (92). Although these scores incorporate nutritional components, they should be interpreted primarily as integrated prognostic indices rather than standalone diagnostic tools for malnutrition. The major nutritional assessment tools, diagnostic frameworks, and integrated prognostic indices that have been applied in patients with hematological malignancies are summarized in Table 2.

**Table 2:** Nutritional assessment tools and integrated prognostic indices applied in hematological malignancies.

Tool	Category	Key components	HM applied population	Main findings in HM
SGA	Nutritional assessment	Weight change, intake, symptoms, physical signs	Leukemia patients after HSCT	SGA identified most leukemia patients after HSCT as undernourished, detecting clinically relevant malnutrition frequently missed by MNA and MUST (148).
PG-SGA	Nutritional assessment	SGA + patient-reported outcomes	HM patients with auto-PBSCT	Longitudinal PG-SGA assessment sensitively captured acute and persistent nutritional impairment in HM patients undergoing auto-PBSCT (86).
GLIM	Diagnostic framework	Phenotypic + etiologic criteria	Hospitalized HM patients	GLIM-defined malnutrition independently predicted increased 1-year mortality in hospitalized HM patients (2).
MNA	Nutritional assessment	Dietary intake questions, BMI, mobility, weight change	Older HM patients	MNA-defined nutritional impairment independently predicted worse overall survival in older patients with HM (87).
CONUT	Integrated prognostic index	Serum albumin, total cholesterol, total lymphocyte count	Hospitalized HM patients	The CONUT score is an independent prognostic factor in patients with HM (89).
mCONUT	Integrated prognostic index	CONUT parameters + CRP	R & R AML patients	The mCONUT is effective for predicting CRS in CLL1 CAR-T therapy (90).
GPS	Integrated prognostic index	CRP, albumin	Hospitalized HM patients	The GPS can predict the length of hospital stay in patients with HM (91).
NII	Integrated prognostic index	NRS-2002, GNRI, SII, LAR(LDH/Alb), β2-microglobulin, CD8^+^ T cells	DLBCL patients	NII score of ≥6 serves as an independent high-risk prognostic factor in DLBCL patients (92).

Anthropometric measurements (skinfold thickness, mid-arm muscle circumference) and bioelectrical impedance analysis (BIA) are used to assess lean body mass and fat composition (93). Previous studies have shown that low subcutaneous adipose tissue at baseline predicts poor survival outcome in patients with MM (94). Reduced standardized phase angle measured via BIA is an independent risk factor for poor survival outcomes in allo-HSCT (95). These advanced metrics are particularly crucial in pediatric patients, where traditional BMI may fail to detect skeletal muscle depletion (96).

Basic laboratory parameters complement physical assessment. Common indicators include serum albumin, transferrin, CRP, serum glucose, and electrolytes (2, 97). Nitrogen balance is also utilized to reflect protein equilibrium, although vomiting and diarrhea in HM patients may compromise its accuracy. Beyond standard panels, specific biomarkers offer targeted insights. Research indicates that retinol-binding protein is specific for autologous HSCT assessment, while transferrin is better suited for allogeneic HSCT (98). Furthermore, while conventional markers like total protein may fail to identify early risk, novel biomarkers such as CXCL13 and GDF15 provide valuable insights for early nutritional assessment in chemotherapy patients (99).

Despite the availability of multiple scoring systems, most were developed in heterogeneous oncologic populations and subsequently extrapolated to HM, raising concerns regarding sensitivity to disease-specific metabolic and inflammatory dynamics. Consequently, the lack of assessment tools specifically developed or prospectively validated for HM patients remains an urgent unmet need.

### 3.3 Comprehensive metabolic and functional evaluation

Following the establishment of malnutrition diagnosis, a comprehensive metabolic and functional evaluation is required to explore the etiology, types, and consequences of malnutrition. This multidimensional investigation encompasses energy expenditure, stress levels, inflammatory response, metabolic status, organ function, and psychological state.

Assessment should be dynamic rather than static. The frequency of evaluation must be determined by the patient’s clinical status and potential body composition changes during hospitalization. For HM patients in the ICU, assessment must extend beyond traditional indicators (weight, BMI) to include disease-specific alterations, organ failure severity, and the need for life support. Ultimately, identifying sarcopenia is critical for formulating optimal individual medical nutrition therapy (MNT) plans.

Such integrative evaluation provides the biological and functional rationale for tailoring the timing, route, and targets of medical nutrition therapy in patients with hematological malignancies.

## 4 Nutritional interventions in hematologic malignancies

The treatment of HM is often accompanied by malnutrition and hypermetabolism in patients. Consequently, systematic nutritional support serves as a crucial adjunctive measure to improve clinical outcomes. However, it should be emphasized that the strength of evidence supporting nutritional interventions in HM varies substantially across modalities and clinical endpoints, with most data derived from observational studies or small interventional trials rather than adequately powered randomized controlled studies. These strategies primarily encompass oral nutritional intervention, enteral nutrition (EN), and parenteral nutrition (PN). Oral nutrition refers to diet-based and supplement-assisted intake by mouth, including dietary counseling and functional food components. Enteral nutrition denotes tube-based medical nutrition therapy administered when oral intake is insufficient or not feasible, while parenteral nutrition is reserved for cases of gastrointestinal failure. The selection and application of these methods require individualized adjustment based on the patient’s specific treatment phase, gastrointestinal function, and nutritional risk.

### 4.1 Oral nutritional interventions and diet-based strategies

Oral nutritional intervention remains the preferred first-line strategy for patients with HMs whenever gastrointestinal function is preserved. Available oral strategies in HM broadly include intensified dietary counseling with oral nutritional supplements (ONS), energy-dense or texture-modified diets, and targeted micronutrient or lipid supplementation (100). Systematic reviews of oral dietary interventions for hematologic neoplasms show mixed results, revealing benefits in some subgroups while overall evidence remains uncertain due to study heterogeneity and limited RCT data (100). The efficacy of these interventions is closely linked to formulation design and individual patient needs, such as chemotherapy-induced taste alterations. A randomized cross-over study demonstrated that high-energy-dense diets effectively increase body weight and BMI by accommodating taste changes during chemotherapy (101). However, this evidence is derived from a small-sample crossover study, and larger randomized trials are warranted to confirm these benefits. Similarly, intensified oral nutrition helps maintain weight stability or promote gain (102). However, a RCT indicated that single-nutrient supplements such as fish oil and glutamine show no substantial benefits for weight, BMI, or anthropometric parameters (103). It should be noted that the interpretation of these negative findings is limited by the heterogeneity of the study population and the lack of standardized dosing protocols. Regarding treatment tolerance, specific interventions offer targeted benefits. Evidence from randomized controlled trials suggests that selenium supplementation may reduce the incidence of severe oral mucositis by activating antioxidant pathways (104), and cooked diets have been associated with a lower risk of bacteremia during chemotherapy (105). However, current guidelines regarding restrictive neutropenic diets are increasingly conflicting, and the evidence supporting their protective role against infection remains weak and largely observational. Similarly, data on selenium efficacy are limited by small cohort sizes. Furthermore, fish oil supplementation may improve long-term survival in leukemia and lymphoma patients by mitigating inflammatory responses (103). However, most interventions—including glutamine and low-bacterial diets—have limited overall benefits regarding core tolerance indicators like neutropenia or hospital length of stay. Currently, no oral nutritional intervention has been confirmed to reduce mortality, suggesting these strategies serve primarily as adjunctive support rather than disease-modifying therapies. This likely reflects the multifactorial nature of adverse outcomes in HM, in which nutritional deterioration represents only one component of a broader inflammatory–metabolic disease process.

Increasing attention has focused on diet-mediated modulation of the gut microbiota as a potential adjunctive strategy, particularly in the HSCT setting. HM treatments often induce dysbiosis, which is linked to complications such as GVHD and infection (106). Observational study indicates that preserving specific bacteria, such as *Eubacterium limosum*, correlates with lower relapse risks after HSCT (107). In a prospective safety and feasibility study, Ladas et al. reported a favorable safety profile of *Lactobacillus plantarum* in pediatric allogeneic HSCT, with no cases of probiotic-associated bacteremia observed (108). While *Lactobacillus rhamnosus GG* may attenuate gastrointestinal side effects in children with acute leukemia (109), other RCT in allo-HSCT patients found no significant impact on microbiota diversity or GVHD incidence (110). Dietary fiber and prebiotic intake appear to exert more consistent microbiome-level effects. In an observational cohort study, D’Angelo et al. reported that higher dietary fiber intake was associated with greater gut microbiota diversity and improved treatment responses in patients with MM undergoing autologous HSCT (111). Shah et al. observed an association between higher intake of dietary flavonoids and plant-derived prebiotics, increased fecal microbiota diversity, and sustained MRD negativity in multiple myeloma survivors receiving lenalidomide maintenance therapy (112). Prebiotic intake was associated with the preservation of butyrate-producing bacteria and a reduced incidence of acute GVHD in allo-HSCT recipients, as reported in a prospective clinical study (113). Despite these positive findings, interindividual differences in microbiota composition, dietary adherence, and variations in fiber types consumed may contribute to heterogeneity across study results. However, the clinical application of probiotics in immunocompromised HM patients remains cautious due to safety concerns regarding potential translocation and bacteremia. Most current evidence is derived from small-scale feasibility studies, and robust efficacy data from large-scale RCTs are still lacking.

### 4.2 Enteral nutrition

When oral intake is insufficient to meet nutritional requirements, enteral nutrition (EN) should be considered as the preferred next-line strategy in patients with preserved gastrointestinal function. Beyond caloric and protein delivery, EN exerts biologically relevant effects on intestinal structure and immune homeostasis, which are particularly important during intensive cytotoxic therapy and HSCT. Continuous enteral nutrient exposure supports intestinal epithelial integrity, limits mucosal atrophy, and preserves gut barrier function, thereby reducing bacterial translocation and systemic inflammatory activation.

Accumulating evidence suggests that EN may favorably influence gut microbiota composition in patients with HM, particularly in the post-transplant setting. Formulations containing glutamine, fiber, and oligosaccharides have been shown to reduce bacterial translocation and significantly improve 100-day survival rates post-HSCT (114). In pediatric HSCT cohorts, EN has been associated with faster recovery of microbial diversity and lower rates of bloodstream infection and acute GVHD (115). Although some adult pilot studies found no significant diversity differences between EN and PN, EN groups still exhibited trends toward higher beneficial bacteria abundance (116).

Comparisons between EN and PN generally favor enteral support. Large-scale retrospective studies indicate that HSCT recipients receiving EN exhibit significantly lower non-relapse mortality and reduced incidence of severe acute GVHD compared to those on PN (117–119). While these findings are clinically significant, they are largely based on retrospective analyses, which may be subject to selection bias. Further prospective validation is required to elevate the strength of this recommendation. Comparative cohort evidence further support this, showing that EN is associated with lower ICU transfer rates and reduced infection-related mortality (120, 121). Additionally, EN significantly reduces hospital length of stay in adults with HM (9). Given the higher risks of metabolic complications and infections associated with PN, EN is recommended as the first-line modality, while PN should be reserved for cases of severe mucositis or gastrointestinal failure (12, 122). Guidelines such as ESPEN recommend a stepwise nutritional strategy: start with counseling and oral supplements, advance to EN if insufficient, and use PN only when EN is not feasible (123).

### 4.3 Parenteral nutrition

For patients undergoing high-dose chemotherapy (HDC) who cannot tolerate enteral feeding, PN can be safely administered via indwelling central venous catheters. Standard PN relies on premixed balanced bags containing amino acids, glucose, lipids, and fluids, supplemented with vitamins and electrolytes. The recommended daily non-protein calorie intake is 25–35 kcal/kg, with 60–70% derived from glucose and 30–40% from intravenous fat emulsions (124). Regular monitoring of fluid balance, glucose, and liver function is essential during administration.

The role of glutamine in PN is controversial. While glutamine-enriched PN (GEPN) may shorten lymphocyte recovery time and reduce mucositis severity in autologous transplant patients (125), other randomized trials indicate that long-term outcomes may be inferior to standard PN, suggesting potential adverse effects (126). While medium-chain triglycerides (MCTs) theoretically offer metabolic advantages over conventional long-chain triglycerides (LCTs), clinical evidence remains equivocal. Some randomized studies suggest LCT-only regimens are more advantageous in reducing febrile neutropenia duration (74). In autologous HSCT, individualized PN—based on calculated energy expenditure—outperforms conventional PN by shortening hospital stays and reducing infection risks (127).

Despite the preference for EN, TPN remains a necessary option for high-risk patients. Recent studies confirm that when used appropriately, TPN effectively mitigates weight loss and improves survival rates (from 2 months to 1 year) without significantly increasing catheter-related infections or liver dysfunction (128).

### 4.4 Nutritional management across the hematopoietic stem cell transplantation continuum

The nutritional status of transplant recipients exerts a pivotal influence on clinical outcomes throughout the HSCT trajectory (129). For patients with HM undergoing HSCT, daily supplementation with whey protein (0.3–0.4 g/kg body weight) plus oral nutritional supplements if needed prior to transplantation, combined with exercise training, can effectively improve health-related quality of life (130). Animal models suggest that a soy-whey protein blend is superior to whey protein alone in promoting hematopoietic and immune reconstitution post-transplant (131). In the early post-transplant period, gastrointestinal toxicity induced by conditioning regimens frequently results in inadequate nutrient intake and malnutrition, while metabolic derangements such as hyperglycemia further exacerbate immune dysfunction and heighten susceptibility to adverse events (132). The goal is to establish individualized caloric intake (25–45 kcal/kg/d), prioritizing EN to maintain mucosal integrity (133). These targets are largely extrapolated from critical care and transplant nutrition guidelines and have not been prospectively validated in HM-specific randomized trials. Insufficient early oral intake (>9 days) correlates with poor prognosis and severe GVHD (134). During long-term follow-up of HSCT recipients, both malnutrition and metabolic syndrome substantially compromise patients’ quality of life and long-term prognosis (135, 136). Routine screening and personalized interventions are necessary to preserve quality of life (137).

Since the advent of HSCT, the neutropenic diet has been routinely implemented in clinical practice based on the theoretical premise of “infection prevention.” This diet strictly restricts foods deemed “potentially microbiologically contaminated”—including fresh fruits and vegetables, raw meat, and unpasteurized dairy products (138). However, current evidence indicates that this diet confers no survival advantage and may even increase infection risks or negatively impact nutritional status (12, 139, 140). Consequently, clinical practice is shifting away from restrictive diets toward emphasizing safe food handling practices (141).

Immunomodulating diets (IMDs) containing arginine, glutamine, and Omega-3 fatty acids theoretically benefit HSCT patients by improving immune function, similar to their effects in surgical patients (142, 143). Specifically, Omega-3 PUFAs (EPA and DHA) may enhance therapeutic efficacy by modulating inflammatory microenvironments (144).

## 5 Challenges and future directions

Despite the established importance of nutritional support in the management of HM, significant barriers impede the translation of current evidence into optimal clinical practice. The heterogeneity of HM pathologies, the complexity of modern therapeutic regimens—including HSCT and CAR-T therapy—and the metabolic distinctiveness of the host response create a landscape where “one-size-fits-all” nutritional strategies are increasingly inadequate. Future progress is likely to benefit from a shift toward precision nutrition, grounded in a deeper mechanistic understanding of host-tumor-nutrient interactions. A critical limitation in current practice is the reliance on caloric supplementation without accounting for the immune-metabolic consequences of specific nutrients. While macronutrient support aims to prevent catabolism, the intersection of nutrient availability and immune effector function remains under-characterized. For instance, preclinical and theoretical models suggest that alterations in amino acid metabolism—specifically the depletion of tryptophan or arginine—may drive T-cell exhaustion, potentially compromising the efficacy of immunotherapies (145). Similarly, the modulation of the gut microbiota represents a promising but immature frontier. While dysbiosis is clearly linked to adverse outcomes like GVHD and infection (106), the precise interaction between nutrient substrates, the microbiome, and immune reconstitution remains a “black box.” Future investigations must utilize metagenomics to tailor interventions, moving beyond generic probiotic supplementation to targeted “biotic” therapies.

This need for precision is currently hampered by the lack of validated assessment instruments specific to the HM population. While tools such as the NRS-2002 and PG-SGA are widely employed (69, 84), they are derived from general oncology or surgical cohorts and may not fully capture the rapid metabolic shifts unique to hematologic crises or conditioning regimens. Furthermore, traditional anthropometrics like BMI are frequently misleading in this population; they fail to detect sarcopenic obesity or the “nutritional inflammatory risk” characterized by preserved weight but depleted muscle mass and elevated inflammatory markers. Consequently, future research should specifically aim to develop and validate HM-specific nutritional assessment tools. These next-generation tools must go beyond traditional metrics by integrating precise body composition analysis (e.g., BIA) with systemic inflammatory markers (e.g., CRP and IL-6). Such a multidimensional approach will better capture the immuno-metabolic vulnerability of patients and guide more personalized interventions.

From a therapeutic perspective, the optimal timing, intensity, and composition of nutritional interventions remain poorly defined due to a scarcity of robust, randomized controlled trials. Clinical practice is currently burdened by conflicting evidence, particularly regarding dietary restrictions. The “neutropenic diet,” historically prescribed to prevent infection, lacks robust evidence supporting a survival benefit and may paradoxically compromise nutritional intake and quality of life (139). The field is increasingly moving away from such restrictive practices toward safe food handling protocols. Similarly, while immune-nutrition (e.g., glutamine, omega-3 fatty acids) theoretically modulates inflammation, clinical trials have yielded equivocal results (142), with some studies even suggesting potential harm or inferior outcomes in specific transplant settings (126). These adverse signals appear to be context-dependent and may vary according to transplant type, conditioning intensity, and baseline nutritional status. Large-scale trials are urgently needed to evaluate clinically meaningful outcomes—such as treatment adherence and toxicity—especially in the context of novel targeted agents and cellular therapies.

A profound gap exists between nutritional guidelines and bedside implementation. Efficacy is frequently compromised by patient adherence issues, psychosocial distress, and a lack of systematic education. Establishing multidisciplinary nutritional support teams (NSTs) as a standard of care is imperative to bridge the implementation gap (6). Research confirms that efficacy-based education enhances self-management capabilities and quality of life, verifying the critical role of educational interventions (146). Furthermore, as survival rates improve, survivorship guidelines must evolve to include long-term monitoring for metabolic sequelae, such as metabolic syndrome and secondary cardiovascular risk, which are emerging as major challenges for survivors. Prospective survivorship cohorts integrating nutritional, metabolic, and cardiovascular outcomes are needed.

Current nutritional protocols are often adult-centric, leaving critical gaps for vulnerable subgroups. Children with HM face the dual burden of fighting malignancy while maintaining growth. Malnutrition in this demographic has severe, lifelong implications for neurodevelopment and physical growth. Future protocols must integrate pediatric-specific screening tools and establish age-appropriate micronutrient standards to prevent developmental stalling during prolonged therapy. For the aging HM population, BMI is a misleading metric that often masks sarcopenic obesity. Future research should prioritize strategies aimed at preserving functional muscle mass through combined nutritional and resistance exercise interventions, targeting the reversal of the “cachexia-frailty” cycle that drives mortality in older patients.

## 6 Conclusion

Malnutrition is a common yet underrecognized determinant of outcome in hematologic malignancies, reflecting a state of integrated immunometabolic vulnerability rather than inadequate caloric intake alone. Tumor-driven hypermetabolism, treatment-induced inflammatory stress, gastrointestinal toxicity, and psychosocial burden converge to impair treatment tolerance, delay immune reconstitution, increase complications, and adversely affect survival across chemotherapy, HSCT, and cellular therapies. From a clinical perspective, validated screening and assessment tools should be systematically embedded within standard care pathways to enable early risk identification, formal diagnosis, and longitudinal monitoring across treatment phases. Although currently applied tools such as NRS-2002, GLIM, and PG-SGA provide a structured and feasible framework for nutritional evaluation, they are largely extrapolated from general populations and lack specificity for the biological heterogeneity and treatment intensity of hematologic malignancies. The development of disease- and treatment-adapted nutritional evaluation tools is therefore warranted to more accurately capture metabolic vulnerability and to guide individualized nutritional interventions. Future nutritional interventions should shift toward mechanism-informed and individualized approaches, incorporating immunometabolic modulation and microbiota-aware strategies alongside standard support. Collectively, nutritional interventions should be integrated as a core component of hematologic cancer management, and advancing precision nutrition through multidisciplinary collaboration represents a tangible opportunity to improve outcomes across the disease continuum.
